# The barriers, facilitators and association of vaccine certificates on COVID-19 vaccine uptake: a scoping review

**DOI:** 10.1186/s12992-023-00969-y

**Published:** 2023-09-27

**Authors:** David T. Zhu, Mohamed Serhan, Salima S. Mithani, David Smith, Joyce Ang, Maya Thomas, Kumanan Wilson

**Affiliations:** 1 Clinical Epidemiology Program, The Ottawa Hospital Research Institute, Ottawa, ON Canada; 2 Medical Scientist Training Program, Virginia Commonwealth University School of Medicine, Richmond, VA USA; 3 School of Epidemiology and Public Health, University of Ottawa, Ottawa, ON Canada; 4 Department of Medicine, University of Ottawa, Ottawa, Canada; 5 Bruyère Research Institute, Ottawa, Canada; 6 O’Neill Institute, Georgetown University, Washington DC, USA

**Keywords:** COVID-19 vaccines, Vaccine certificates, Barriers, Facilitators

## Abstract

**Background:**

Globally, COVID-19 vaccines have proven to be instrumental for promoting population health by reducing illness from SARS-CoV-2. Vaccine certificates emerged as a potentially promising solution for encouraging vaccination and facilitating the safe reopening of society, however, they were controversial due to criticisms of infringing upon individual rights. While there is extensive literature describing the ethical, legal, and public health implications of vaccine certificates, there is currently a gap in knowledge about the association of vaccine certificates on vaccine uptake during the COVID-19 pandemic and barriers and facilitators to their use.

**Objectives:**

The objectives of this scoping review are to (i) describe the existing literature on the association of vaccine certificates on the rates of COVID-19 vaccine uptake across several countries and (ii) describe the intrinsic and extrinsic barriers or facilitators that moderate this relationship.

**Methods:**

We conducted a scoping review based on PRISMA Extension for Scoping Reviews (PRSIMA-ScR) guidelines. We searched three bibliographic databases (APA PsychInfo, Embase Classic + Embase, OVID-Medline) and preprint severs during the first week of July 2023. Three reviewers independently screened the studies based on pre-specified eligibility criteria and performed quality assessments of the primary literature and data extraction.

**Results:**

Sixteen studies met the inclusion criteria. 14 or these were surveys and 2 were modelling studies. The majority documented that vaccine certificates were significantly associated with increased rates of COVID-19 vaccine uptake (*n* = 12), motivated by factors such as travel/employer requirements, influence from the government/peers, and trust in the safety, efficacy, and science behind COVID-19 vaccines. Three studies had non-significant or mixed findings. Only one study found a significant decrease in COVID-19 vaccine uptake, motivated by pervasive distrust in the QR code-based system of digital vaccine certificates in Russia. Quality of survey studies was generally high.

**Conclusion:**

Our findings provide insights into the existing literature on vaccine certificates association with vaccine uptake in several different jurisdictions and barriers and facilitators to their uptake. This information can be used to guide future examinations of the implementation of vaccine certificates and more effective implementations.

**Supplementary Information:**

The online version contains supplementary material available at 10.1186/s12992-023-00969-y.

## Introduction

Globally, governments implemented public health measures, including quarantine/stay at home orders, social distancing, lockdowns and closures of various social or commercial venues, travel restrictions, vaccination, and more, to help mitigate the impact of the SARS-CoV-2 virus [1, 2]. Vaccination is critical to protecting the public from the deleterious health consequences of COVID-19 infection and facilitating the reopening of the economy and society at-large [3–6].For the latter, vaccine certificates have been introduced as a hybrid approach of gating access to certain privileges (e.g., cross-border travel, return to work, access to certain shared public spaces and venues, etc.) under the condition of vaccination against COVID-19 [7–10]. However, a major criticism, from a human rights and ethical perspective, is that vaccine certificates infringe individual rights and freedoms, particularly their right to bodily autonomy [11–14]. Further, several upstream social determinants, including influence from friends, family, sources of information, and more, impact one’s willingness to vaccinate and obtain vaccine certificates [15–17]. This issue is further complicated by the fact that different countries introduced different types of vaccine certificates, using different approaches, and at different timelines [18, 19]. For example, it was found that in certain regions, such as the European Union, vaccination certificates served as a means to gate international travel. However, in China and the United States of America, they were more commonly used for gating access to activities of daily life within the country. In Canada, India, South Africa, Korea and the United Kingdom, vaccine certificates were used for gating access to activities of daily life and international travel [20]. Altogether, it is not yet clear how vaccine certificates may be associated willingness to vaccinate and whether these effects vary across various settings and timelines. To address this gap in the literature, we have conducted a scoping review to investigate the association between vaccine certificates and willingness to vaccinate against COVID-19 and barriers and facilitators to their impact.

## Methods

### Objectives

The objectives of this scoping review are to (i) describe the existing literature on the association of vaccine certificates on the rates of COVID-19 vaccine uptake across several countries and (ii) describe the intrinsic and extrinsic barriers or facilitators that moderate this relationship.

### Methodological approach

We conducted a structured scoping review in accordance with PRISMA Extension for Scoping Reviews (PRISMA-ScR) guidelines to identify and describe both peer-reviewed and grey literature within the topic of COVID-19 vaccine certificates and COVID-19 vaccine hesitancy.

A scoping review was conducted given the expected heterogeneity of the primary data. Implementations of vaccine certificates varied significantly across the globe limiting the ability to draw strong conclusions from a formal evidence synthesis [21]. However, substantial value would be garnered from capturing the range and breadth of the studies on this important intervention.

We followed the Preferred Reporting Items for Systematic reviews and Meta-Analyses extension for Scoping Reviews (PRISMA-ScR) Checklist (Appendix 2).

### Information sources and search strategy

Three bibliographic databases (APA PsychInfo, Embase Classic + Embase, OVID-Medline) were searched for published, peer-reviewed literature, and three repositories (Medrxiv, Biorxiv, L·OVE) were searched to identify pre-print records on the first week of July 2023 for articles related to Covid-19 and vaccine certificates (search terms are included in Appendix 1). The search strategy was co-developed and executed by an experienced medical librarian. A detailed description of the search strategy, including combinations of MeSH terms, can be found in supplementary document 1. The inclusion and exclusion criteria in this study can be found in supplementary document 2.

### Eligibility criteria

For articles to have been included in this review, they must have met the following criteria:

• Examined the general adult population rather than special/vulnerable populations.
• Included discussion of COVID-19 vaccine certificate characteristics (or synonyms such as immunity passports, green passes, proof of vaccination, etc.)
• Included discussion of participants’ willingness to receive COVID-19 vaccines (acceptance, delay, ambivalence, hesitancy, etc.)
• Evaluated the potential role of COVID-19 vaccine certificate on willingness to vaccinate
• Available in English
• Considered primary research

### Selection of sources of evidence

Titles, abstracts, and relevant full texts of retrieved records were screened by three independent reviewers (DZ, JA, MT) based on pre-specified inclusion and exclusion criteria (Tables 1 and 2). Any conflicts that arose during screening were resolved by a neutral third reviewer (SSM, and MS) who was not involved in the initial review of the papers.

**Table 1:** Eligibility criteria

The following articles were included:
Examined the general adult population rather than special/vulnerable populations Included discussion of COVID-19 vaccine certificate characteristics (or synonyms such as immunity passports, green passes, proof of vaccination, etc.) Included discussion of participants’ willingness to receive COVID-19 vaccines (acceptance, delay, ambivalence, hesitancy, etc.) Evaluated the potential role of COVID-19 vaccine certificate on willingness to vaccinate English language full text available Primary research (e.g., observational, modeling, experimental, and qualitative studies)
**The following articles were excluded:**
No discussion of COVID-19 vaccine certificates (e.g., studies that broadly mentioned “vaccine mandates” without specifying vaccine certificates or synonyms) No discussion of COVID-19 vaccine intention or uptake Studies that generally described public opinions and attitudes on COVID-19 certificates and/or hesitancy but did not evaluate their interaction/association No English language full text available Review-type or non-empirical studies (e.g., commentaries, editorials, opinion letters, etc.)

**Table 2:** Quality of Studies

References	Was a clear research question posed?	Was the target population defined, and was the sample representative of the population?	Was a systematic approach used to develop the questionnaire?	Was the questionnaire tested?	Were questionnaires administered in a manner that limited both response and nonresponse bias?	Was the response rate reported, and were strategies used to optimize the response rate?	Were the results clearly and transparently reported?	Comments
Determinants and variations of COVID-19 vaccine uptake and responses among minority ethnic groups in Amsterdam, the Netherlands [22]	TRUE	TRUE	FALSE	FALSE	TRUE	TRUE	TRUE	Did not state if survey was tested or specify testing/development process of questionnaire used
Public policy measures to increase anti-SARS-CoV-2 vaccination rate in Russia [23]	TRUE	TRUE	FALSE	FALSE	TRUE	TRUE	TRUE	Did not state if survey was tested or specify testing/development process of questionnaire used
Persistence of vaccine hesitancy and acceptance of the EU Covid certificate among French students [24]	TRUE	TRUE	TRUE	FALSE	TRUE	TRUE	TRUE	Target population was defined, but representativeness of the sample was described in the paper as a limitation (i.e. surveyed students who were hesitant about vaccines against Covid-19 until very recently and who made their decision to be vaccinated later than their peers) Did not mention if survey was tested
The role of incentives in deciding to receive the available COVID-19 vaccine in Israel [25]	TRUE	TRUE	FALSE	TRUE	TRUE	TRUE	TRUE	Target population was defined, but representativeness of the sample was described in the paper as a limitation (i.e. due to use of online recruitment methods, may not be representative of entire Israeli population/inclusive of minorities). Questionnaire was partly based on a previous questionnaire that was pilot-tested within the general public in June 2020
COVID-19 vaccine hesitancy in a city with free choice and sufficient doses [26]	TRUE	FALSE	TRUE	TRUE	TRUE	TRUE	TRUE	
The potential impact of vaccine passports on inclination to accept COVID-19 vaccinations in the United Kingdom: Evidence from a large cross-sectional survey and modeling study [27]	TRUE	TRUE	FALSE	FALSE	TRUE	TRUE	TRUE	Did not state if survey was tested or specify testing/development process of questionnaire used
COVID-19 vaccination mandates and vaccine uptake [28]	TRUE	TRUE	TRUE	FALSE	TRUE	TRUE	TRUE	Did not explicitly state if the survey was tested
The effect of COVID certificates on vaccine uptake, health outcomes, and the economy [29]	TRUE	TRUE	FALSE	FALSE	TRUE	FALSE	TRUE	“Global COVID-19 Trends and Impact Survey” in partnership with Facebook—the questionnaire used to obtain survey data; the target population is unclear
The effect of mandatory COVID-19 certificates on vaccine uptake: synthetic-control modelling of six countries [30]	TRUE	TRUE	TRUE	TRUE	TRUE	TRUE	TRUE	Does not explicitly state if the survey was tested
United States COVID-19 vaccination preferences (CVP): 2020 hindsight [31]	TRUE	TRUE	TRUE	TRUE	FALSE	TRUE	TRUE	Online survey (underrepresents those who are unable to use technology or are illiterate), but target population may be considered geographically and demographically representative
COVID-19 vaccine hesitancy in eight European countries: prevalence, determinants, and heterogeneity [32]	TRUE	TRUE	TRUE	FALSE	TRUE	TRUE	TRUE	Did not explicitly state if the survey was tested
COVID-19 vaccine hesitancy and vaccine passports: a cross-sectional conjoint experiment in Japan [32]	TRUE	TRUE	TRUE	FALSE	TRUE	TRUE	TRUE	Did not explicitly state if the survey was tested
Public attitudes to COVID-19 vaccines: a qualitative study [33]	TRUE	TRUE	TRUE	FALSE	FALSE	TRUE	TRUE	People aged 50 + years were underrepresented in the final sample, participants met in online focus groups which could impact their responses. Did not explicitly state if the survey was tested
Exploring the impact of Quebec's vaccine lottery and vaccine passports on Covid-19 vaccination intention: Findings from repeated cross sectional surveys [34]	TRUE	TRUE	FALSE	FALSE	TRUE	TRUE	TRUE	Did not state if survey was tested or specify testing/development process of questionnaire used
Carrot-and-Stick, or Piling Coffins? Estimating the Role of Factors Overcoming COVID-19 Vaccine Hesitancy in Poland and Lithuania in the Years 2021–2022 [35]								N/A, no survey was used in this study and instead a weighted regression model was used
The effect of vaccine mandate announcements on vaccine uptake in Canada: An interrupted time series analysis [36]								N/A, no survey was used in this study and instead a time series analysis was conducted

### Data charting process and items

Included full text articles that remained after screening then underwent data extraction by 3 reviewers (DTZ, JA, MT), with 2 other independent reviewers performing verification (SSM, MS). Preprint studies remaining after screening were updated with the final peer-reviewed publication if available.

Information was collected and inputted into a data extraction form with prespecified categories. Data extraction endpoints included reference details (author, publication year, study design, location, period of data collection), study population characteristics (demographic information, proportion of vaccinated/unvaccinated), methods of recruitment and assessment (surveys, scales, interviews, etc.), details about the intervention/experimental design (if applicable), theoretical frameworks/models used, vaccine passport/certificate characteristics (types of vaccines, digital technologies used, public attitudes/opinions towards vaccine certificates, reason(s) for seeking a vaccine certificate), and vaccination intention (acceptance, delay, ambivalence, hesitancy). The 3 C’s Model of Vaccine Hesitancy was used to categorize barriers to vaccination [21].

### Analysis, synthesis, and presentation of results

Studies were analyzed according to article characteristics (i.e., article type such as qualitative, survey, quantitative modelling, etc.), assessment of themes and subthemes, and various elements of content, and the observed association of vaccine certificates on willingness to vaccinate. Articles were then grouped based on study design (i.e., observational, modeling, experimental, and qualitative designs), barriers and facilitators of willingness to vaccinate, and overall association of vaccine certificates on the rates of vaccine uptake.

The quality of all included surveys was assessed using 7 different criteria [37]. 1) Was a clear research question posed? 2) Was the target population defined, and was the sample representative of the population? 3) Was a systematic approach used to develop the questionnaire? 4)Was the questionnaire tested? 5)Were questionnaires administered in a manner that limited both response and nonresponse bias? 6)Was the response rate reported, and were strategies used to optimize the response rate? 7) Were the results clearly and transparently reported? [38].

## Results

### Selection of studies

**Fig. 1:**
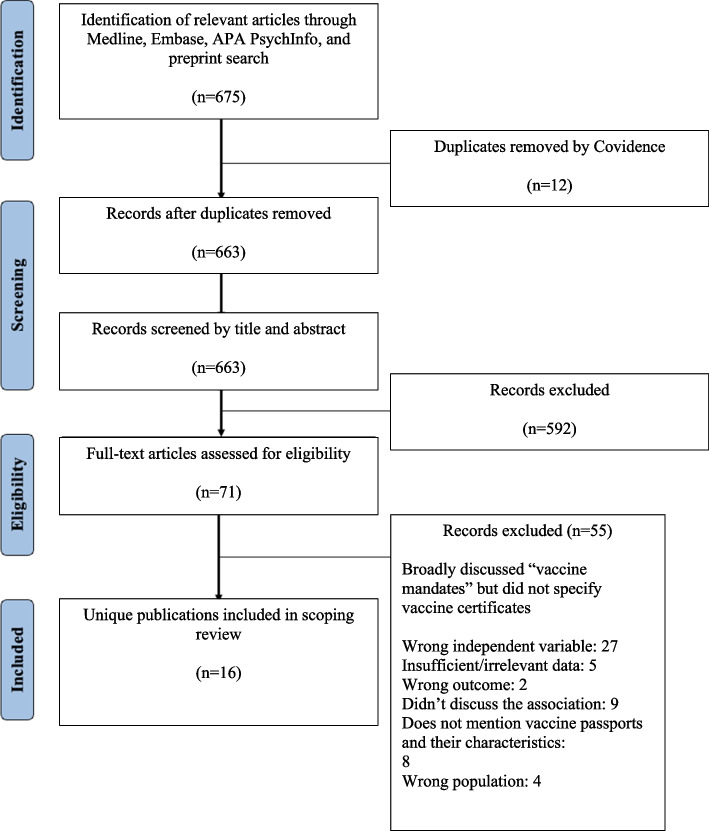
PRISMA flow diagram

Our search strategy initially identified 675 articles. After duplicates (*n* = 12) were automatically removed by Covidence, title and abstract screening resulted in the exclusion of 592 articles from 663 articles, and full-text screening resulted in the exclusion of an additional 55 articles. The remaining 16 articles are included in the manuscript. A comprehensive overview of the screening process is presented in a PRISMA flowchart (Fig. 1).

### Article types and general characteristics

Of the 16 total studies included, there were four main types of study designs: observational (*n* = 8), modeling (*n* = 4), experimental (*n* = 3), and qualitative (*n* = 1) (Table 3).

**Table 3:** General characteristics of included studies

Publication	Country	Study Design	Data Collection Period	Data Collection Method	Theoretical Framework
Determinants and variations of COVID-19 vaccine uptake and responses among minority ethnic groups in Amsterdam, the Netherlands [22]	Netherlands	Observational	Jan 30, 2021 – April 30, 2021	Data about personal characteristics and COVID-19 vaccine intentions collected via an online survey (Google Forms) or paper questionnaire; virtual follow-up interviews were conducted on a subset	3 C’s Model of Vaccine Hesitancy
Public policy measures to increase anti-SARS-CoV-2 vaccination rate in Russia [23]	Russia	Observational	Sept 1, 2021 – Jan 25, 2021	Data about attitudes towards COVID-19 vaccination and QR code-based vaccine certificates collected via VKontakte social network and Silverlight applet	Unspecified
Persistence of vaccine hesitancy and acceptance of the EU Covid certificate among French students [24]	France	Observational	Sept 13, 2021 – Sept 24, 2021	Data about personal characteristics and attitudes towards COVID-19 vaccines and the EU COVID Certificate collected via in-person surveys and for a subset, interviews at a mobile vaccine clinic	Health Belief Model
The role of incentives in deciding to receive the available COVID-19 vaccine in Israel [25]	Israel	Observational	Dec 22, 2021 – Jan 10, 2021	Data about personal characteristics and attitudes towards incentives to obtaining COVID-19 vaccination collected via an online survey (Google Forms)	Health Belief Model
COVID-19 vaccine hesitancy in a city with free choice and sufficient doses [26]	China	Observational	Apr 23, 2021 – May 8, 2021	Data about personal characteristics, COVID-19 vaccine intentions, and pandemic attitudes collected via telephone interview system (TIS)	Unspecified
The potential impact of vaccine passports on inclination to accept COVID-19 vaccinations in the United Kingdom: Evidence from a large cross-sectional survey and modeling study [27].	United Kingdom	Modeling	Apr 9, 2021 – Apr 27, 2021	Data about COVID-19 vaccination intentions and incentives, and vaccine certificates, were collected via a national survey	Unspecified
COVID-19 vaccination mandates and vaccine uptake [28]	Canada, France, Germany, Italy	Modeling	June 15, 2021 – Oct 31, 2021	Data about COVID-19 cases, deaths, and vaccination rates, collected from official provincial dashboards (Canada) and Our World in Data site	Unspecified
The effect of COVID certificates on vaccine uptake, health outcomes, and the economy [29]	France, Germany, Italy	Modeling	Jan 2022	Data about hospital admissions, COVID-19 vaccine uptake, and health economics trends were collected from Our World in Data site, ECDC, INED, OECD Weekly Tracker, and publicly available surveys	Innovation Diffusion Theory
The effect of mandatory COVID-19 certificates on vaccine uptake: synthetic-control modelling of six countries [30]	Denmark, France, Germany, Israel, Italy, Switzerland	Modeling	Apr 21, 2021 – Nov 8, 2021	Data about hospital admissions, COVID-19 vaccine uptake, and government interventions were collected from Our World in Data site, Oxford COVID-19 Government Response Tracker, and ECDC	Unspecified
United States COVID-19 vaccination preferences (CVP): 2020 hindsight [31]	United States	Experimental	Nov 9, 2020 – Nov 11, 2020	Participant choices in this discrete choice experiment were collected via surveys	Health Preference Research
COVID-19 vaccine hesitancy in eight European countries: prevalence, determinants, and heterogeneity [39]	Bulgaria, France, Germany, Italy, Poland, Spain, Sweden, United Kingdom	Experimental	Apr 2021 (Germany) Jun 2021 (all other countries)	Participant choices in this randomized controlled experiment collected via an online survey (Respondi)	Unspecified
COVID-19 vaccine hesitancy and vaccine passports: a cross-sectional conjoint experiment in Japan [32]	Japan	Experimental	Jul 2021 – Nov 2021	Participant choices in this conjoint experiment collected via an online and follow-up survey	Health Belief Model
Public attitudes to COVID-19 vaccines: a qualitative study [33]	United Kingdom	Qualitative	Mar 15, 2021 – Apr 22, 2021	Focus groups were conducted on a virtual conferencing platform (Zoom)	Continuum of Vaccine Hesitancy Model
Exploring the impact of Quebec's vaccine lottery and vaccine passports on Covid-19 vaccination intention: Findings from repeated cross-sectional surveys [34]	Canada	Observational	July 9, 2021—September 1 2021	Invitation to fill out questionnaire was sent via email	5 C’s Model of Vaccine Hesitancy
More Time, Carrot-and-Stick, or Piling Coffins? Estimating the Role of Factors Overcoming COVID-19 Vaccine Hesitancy in Poland and Lithuania in the Years 2021–2022 [35]	Poland and Lithuania	Observational	May 31, 2021—May 29, 2022	Data was collected via the ECDC website	Unspecified
The effect of vaccine mandate announcements on vaccine uptake in Canada: An interrupted time series analysis [36]	Canada	Observational	July—November 2021	Data were drawn from the Canadian COVID-19 Vaccination Coverage Surveillance System (CCVCSS), and sourced from provincial/territorial immunization registries. COVID-19 weekly case, hospitalization and mortality data were obtained from the National COVID-19 Case dataset	Unspecified

### Observational studies

The eight observational studies were all based on a cross-sectional design and spanned eight different countries (i.e., Netherlands, Russia, France, Israel, China, Canada, Lithuania and Poland). Six studies focused on the general adult population within their respective countries [23, 25, 26, 34–36], whereas the others focused on specified subpopulations, including ethnic/racial minorities ^30 ^and university students^31^. These studies involved similarly low rates of COVID-19 vaccination at baseline — namely, 2–14% [22], 17.28% [23] 10.1–13.5% [26], and 3–4% [35] — except for a study conducted immediately after the COVID-19 vaccines were made available, therefore, had no vaccinated participants at baseline [25], and a study that recruited only participants that had received their vaccination dose(s) at a specified vaccine clinic, therefore, all participants were vaccinated at baseline [24]. Another study had an 87% vaccination rate (defined as 2 doses) at baseline [34]. Another study had an average vaccination rate among all provinces at 82.01% at baseline [36]. Two studies used the Health Belief Model as their theoretical framework [24, 25], one study used the 5 C’s Model of Vaccine Hesitancy [34] and one used the 3 C’s Model of Vaccine Hesitancy [22].

### Modeling studies

The four modeling studies primarily examined countries in Europe (e.g., France, Italy, Germany, Denmark, Switzerland, and the United Kingdom) as well as a few non-European countries (e.g., Canada, Israel, and the United States). Only one of these studies used a theoretical framework — innovation diffusion theory — which describes how innovations (particularly, vaccines) are spread and taken up; specifically, in this study, innovation diffusion theory was used to establish the counterfactual estimates [29]. Data on COVID-19 cases, deaths, hospital admissions, vaccination rates, and more, were collected from multinational databases (e.g., Our World in Data, European Centre for Disease Prevention and Control, Oxford COVID-19 Government Response Tracker, etc.) to generate the predictive models [27–30]. All four modeling studies examined trends in the general adult population in their respective countries and were not narrowed to specific subpopulations. The time period these studies took place ranged from one month — April 2021 [27]or December 2021 [29]— to several months in length, July 2021–October 2021 [28] or April 2021–November 2021 [30].

### Experimental studies

The three experimental studies primarily examined Japan [32], the United States [31], and several European countries [39], and were all focused on the general adult population rather than any specific subpopulations. The theoretical frameworks used were the Health Belief Model [32] and Health Preferences Research (HPR) [31]. These experimental studies evaluated the effects of varying characteristics (varying levels efficacy, side effects, settings, presence of vaccine certificates, etc [32]), knowledge translation (messaging about COVID-19 risk reduction, vaccine certificates, and hedonistic or altruistic benefits [39]), and incentives (incentives such as access to travel, restaurants, social gatherings, and going out without masks) associated with vaccine certificates, and to what extent they influenced willingness to vaccinate. These experimental studies used various experimental designs such as a conjoint experimental design [32], a randomized control design [39] and a discrete choice design [31].

### Qualitative studies

Only one qualitative study was captured [33], which focused on examining public attitudes towards COVID-19 vaccines in the United Kingdom. The main questions in the focus groups covered themes such as vaccination intention, perceptions on vaccine certificates, and other vaccine-related experiences and behaviors. The authors employed the Continuum of Vaccine Hesitancy Model as their theoretical framework, which treats willingness to vaccinate as a continuum between complete acceptance and complete refusal [33]. This study was conducted between March 2021–April 2021.

### Quality assessment

Fourteen out of 16 studies in this review had used a survey. All 14 studies posed a clear research question, indicating a focus on specific objectives. 13 studies met the criterion of defining the target population and ensuring sample representativeness. 8 studies used a systematic approach to develop the questionnaire and 8 studies were found to have followed a systematic process to construct their survey instruments. In terms of administering questionnaires, 12 studies employed methods that aimed to limit both response and nonresponse bias, indicating an effort to collect accurate and unbiased data. Additionally, 13 studies reported their response rates and discussed strategies used to optimize response rates. All 14 studies presented their results clearly and transparently.

### Barriers and facilitators to COVID-19 vaccination

(Table 4) describes major themes in motivation to refuse (“barriers”) or accept (“facilitators”) COVID-19 vaccination. These motivations were further categorized into external influences on vaccination, such as family, friends, community, and other structural influences (“extrinsic barriers and facilitators”) or intrinsic influences on vaccination, such as personal goals, values, concerns, and belief systems (“intrinsic barriers and facilitators”). The 3 C’s model of vaccine hesitancy was also incorporated into our analysis [40].

**Table 4:** Facilitators and barriers to vaccination uptake

Determinant	*n* (%)	References
**Extrinsic barriers to vaccination**		
Privacy concerns over COVID-19 vaccine certificates	2 (12.5%)	[25, 33]
Ethical concerns over COVID-19 vaccine certificates	2 (12.5%)	[25, 33]
Technological concerns over COVID-19 vaccine certificates	1 (6.3%)	[25]
Lack of COVID-19-related information (or misinformation and conspiracy theories)	7 (43.8%)	[24–26, 31–34]
Lack of convenience/accessibility to COVID-19 vaccination	6 (37.5%)	[22–24, 26, 32, 33]
**Intrinsic barriers to vaccination**		
Distrust/lack of confidence in government leaders/policies	5 (31.3%)	[23–25, 31, 33]
Distrust/lack of confidence in public health and pharmaceutical leaders	2 (12.5%)	[23, 24]
Distrust/lack of confidence in the quality/safety of COVID-19 vaccines	8 (50%)	[23–26, 31–33, 39]
Distrust/lack of confidence in the efficacy of COVID-19 vaccines	5 (31.3%)	[23, 24, 26, 32, 33]
Complacency: perception that COVID-19 poses no serious health risks	3 (18.8%)	[23, 31, 39]
Complacency: perception that COVID-19 vaccines are unnecessary/not important	5 (31.3%)	[23, 26, 31, 33, 39]
**Extrinsic facilitators to vaccination**		
Travel requirements	4 (25%)	[23, 24, 33, 39]
Employer requirements	4 (25%)	[23, 24, 26, 29]
Influence from government	3 (18.8%)	[22–24]
Influence from family or friends	3 (18.8%)	[23, 24, 26]
Influence from monetary incentives	2 (12.5%)	[34, 35]
Influence from doctors	1 (6.3%)	[24]
Influence from media	0 (0.0%)	N/A
Influence from other sources	2 (12.5%)	[24, 35]
To help reopen the economy and society	4 (25%)	[24, 29, 33, 39]
**Intrinsic facilitators to vaccination**		
Trust/confidence in the quality/safety of COVID-19 vaccines	2 (12.5%)	[23, 25]
Trust/confidence in the efficacy of COVID-19 vaccines	2 (12.5%)	[23, 24]
Trust/confidence in the science behind COVID-19 vaccines	1 (6.3%)	[33]
Desire to protect themselves	2 (12.5%)	[26, 35]
Desire to protect family and friends	1 (6.3%)	[26]
Desire to protect society	2 (12.5%)	[24, 26]
Perceived moral responsibility	0 (0.0%)	N/A
Convenient/accessible vaccine clinics	2 (12.5%)	[22, 26]

### Extrinsic barriers to vaccination

Privacy concerns were brought up in two studies [23, 33] with regards to themes such as fears of total digitalization, accumulation of digital information in government databases, possible fraud, lack of financial protection (e.g., some Russian banks have integrated digital vaccine certificates into online banking systems), protection especially for children who are issued digital vaccine certificates, and the perceived “Orwellian” nature of vaccine certificates. Technological concerns were mentioned in one study [23] and were closely related to privacy concerns, such as pervasive public distrust of the digital infrastructure underlying vaccine certificates (e.g., distrust of QR code system). Ethical concerns were discussed in two studies [23, 33] and centered around the idea that vaccine certificates, from a human rights perspective, restrict personal autonomy and freedoms such as gatekeeping access to many shared public spaces or social events, travel across borders, employment, and many other privileges. Lack of reliable sources of COVID-19 information, or exposure to COVID-19 misinformation and conspiracy theories, were mentioned in five studies [24, 26, 31, 33, 34] and discussed themes such lack of information about the safety of efficacy of COVID-19 vaccines, lack of information about the short- and long-term side effects of COVID-19 vaccines, and exposure to COVID-19 vaccine-related conspiracy theories or “echo chambers”. Finally, the lack of convenience and accessibility were cited in six studies [22, 24–26, 31, 33], such as barriers to accessing COVID-19 vaccine centers or the unavailability of specific brands (e.g., some are willing to accept particular vaccine brands, but not others).

### Intrinsic barriers to vaccination

Distrust and lack of confidence in certain aspects of COVID-19 vaccines, or towards specific social institutions, were frequently mentioned themes. This distrust and lack of confidence were (a) directed towards government leaders in four studies [22, 23, 26, 33] with regards to themes such as vaccines and vaccine certificates serving as agents of social control; (b) directed towards public health or pharmaceutical agencies in two studies [22, 26] with regards to themes such as a lack of trust in the sources, manufacturers, and countries of origins of COVID-19 vaccines; (c) directed towards the quality and safety of COVID-19 vaccines in seven studies [22–24, 26, 31, 33, 39], focusing on themes such as concerns about potential adverse events from COVID-19 vaccines (particularly long-term side effects), belief that COVID-19 vaccines were developed too quickly and did not undergo sufficient safety testing, belief that COVID-19 vaccines contain harmful substances, a lack of trust in vaccine research and the refusal to be used as a “guinea pig” in vaccine research. Further, complacency was frequently cited as a barrier to vaccination. Specifically, complacency (a) with respect to the perception that COVID-19 is not a serious illness (e.g., “just like the flu”) and does not pose a threat to health and wellbeing was cited in three studies [22, 32, 39]; and (b) with respect to the perception that COVID-19 vaccines are unnecessary since alternative forms of precautions and protection are sufficient to prevent COVID-19 infection and sequelae (e.g., personal protective equipment, masks, natural immunity, and herd immunity) were cited in five studies [22, 24, 32, 33, 39].

### Extrinsic facilitators to vaccination

Discourse about vaccine certificate-mediated privileges centered around travel and employment. Seeking COVID-19 vaccines and vaccine certificates to facilitate both regional and international travel were cited in four studies [22, 26, 32, 33]. Seeking COVID-19 vaccines and vaccine certificates to satisfy employer recommendations or mandates were cited in four studies [22, 24, 26, 27]. We also identified six external sources of influence regarding vaccination: (a) high levels of trust in the government and mandates facilitated vaccination in three studies [22, 26, 39]; (b) influence from the government via monetary incentives facilitated vaccination in two studies [34, 35]. (c) recommendations from friends or family to get vaccinated also predicted increased willingness to vaccinate in three studies [22, 24, 26]; (d) recommendations from physicians and other healthcare providers to get vaccinated led to increased vaccination in one study [26]; (e) influence from the media was not identified to be a facilitator to vaccination in any of the included studies; and (f) influence from other sources was mentioned in one study [26], which discussed the provision of medical absences to allow time for vaccination and relaxing mandatory post-vaccination isolation measures predicted increased uptake of COVID-19 vaccines. Accepting the COVID-19 vaccine to help reopen the economy and society was cited by four studies [26, 27, 32, 33], such as reopening access to various shared public spaces and social events, entertainment venues, religious venues, school venues, restaurants, not needing to use face masks, and more.

### Intrinsic facilitators to vaccination

Trust was a recurring theme in efforts to increase willingness to vaccinate. We identified three main aspects of trust: (a) trust and confidence in the safety and quality of COVID-19 vaccines were cited in two studies [22, 23], with a small number of participants describing how, if currently available vaccines did not meet their safety or quality expectations, then they will wait until a different or foreign-produced vaccine is made available; (b) trust and confidence in the efficacy of COVID-19 vaccines were cited in two studies [22, 26], with many vaccine acceptors believing that vaccination is the most effective strategy to end the COVID-19 pandemic; and (c) trust in COVID-19 vaccine research was cited in one study [24](e.g., although some vaccine acceptors were concerned at the speed at which COVID-19 vaccines had been developed, they rationalized this in terms of science being more advanced nowadays and having more rigorous scientific and financial focus on vaccine development during the COVID-19 pandemic). A desire to protect themselves and others were also recurring themes that predicted increased vaccination uptake. Specifically, (a) the desire to protect themselves was cited in two studies [24, 34], driven by the perception that COVID-19 is a serious illness for which vaccines could reduce the onset, severity, and potential sequelae or complications; (b) the desire to protect family and friends from COVID-19 transmission was cited in one study [32]; and (c) the desire to protect society at-large was cited by two studies [24, 26], driven by a perceived civil responsibility to contribute to herd immunity and protect others in society. Convenience and accessibility of vaccine clinics increased willingness to vaccinate in two studies [24, 25]. Finally, perceived moral responsibility was also evaluated, although none of the included studies mentioned this theme.

### Association of COVID-19 vaccine certificates on willingness to vaccinate

Overall, 12 (75%; *n* = 12/16) reported that COVID-19 vaccine certificates were associated with increased vaccine uptake across multiple countries (Table 5) [22, 24, 26, 28–36].

**Table 5:** Impact of COVID-19 vaccine certificates on willingness to vaccinate

Source	Outcome	Magnitude	Additional Details
*Increased willingness to vaccinate (n* = *12)*			
[31]	Increase in log-odds of COVID-19 vaccination if vaccine certificates were made available in the U.S. (among *total* survey respondents)	0.318 (95% CI: 0.254–0.381)	Subgroup analysis: vaccine certificates increased vaccine uptake *among respondents who want one* by log-odds of 0.383 (95% CI: 0.304–0.463), but did not affect uptake *among respondents who do not want certificates* (log-odds: –0.049; 95% CI: –0.160; 0.258)
[22]	Increase in prevalence of survey respondents “willing to accept” the COVID-19 vaccine if government-mandated vaccine certificates were implemented (in the Netherlands)	22.1 p.p	Prevalence increased from 47.5% (baseline) to 69.6% (if vaccine certificates were implemented)
[28]	Increase in prevalence of COVID-19 vaccine uptake (first doses) after government-mandated vaccine certificates to access to public and non-essential business venues		Increases in vaccination rate compare “actual” vs. “counterfactual” estimates
	Germany	4.7 p.p. (90% CI: 4.1–5.1 p.p.)	Recorded at 11 weeks post-announcement; equivalent to 3.47 (90% CI: 3.06–3.81) million new first doses
	Canada	Up to 5 p.p. (90% CI: 3.9–5.8 p.p.)	Recorded at 5–13 weeks post-announcement across provinces; equivalent to 979,000 (90% CI: 425,000–1,266,000) new first doses
	France	8 p.p. (90% CI: 4.3–10.8 p.p.)	Recorded at 16 weeks post-announcement; equivalent to 4.59 (90% CI: 2.47–6.25) million new first doses
	Italy	12 p.p. (90% CI: 5–15.1 p.p.)	Recorded at 14 weeks post-announcement; equivalent to 6.48 (90% CI: 2.67–8.14) million new first doses
[29]	Increase in prevalence of COVID-19 vaccine uptake (first doses) after government-mandated vaccine certificates to access to public and private venues		Increases in vaccination rate are comparing “actual” vs. “counterfactual” estimates
	Germany	6.2 p.p. (95% CI: 2.6–6.9 p.p.)	Prevented an estimated 1,133 deaths and GDP loss of €1.4 billion in Germany
	Italy	9.7 p.p. (95% CI: 5.4–12.3 p.p.)	Prevented an estimated 1,331 deaths and GDP loss of €2.1 billion in Italy
	France	13.0 p.p. (95% CI: 9.7–14.9 p.p.)	Prevented an estimated 3,979 deaths and GDP loss of €6.0 billion in France
[32]	Prevalence difference in survey respondents “intending to accept” COVID-19 vaccination if vaccine certificates helped relax all public health restrictions, compared to no relaxation of restrictions (in Japan)	27%	Among vaccine-hesitant respondents, 45% intended to get vaccinated if vaccine certificates relaxed COVID-19 public health restrictions, compared to 18% if they are not relaxed
	Increase in prevalence of survey respondents “intending to accept” COVID-19 if vaccine certificates helped relax specific restrictions (in Japan):		
	Travel across prefectures	10 p.p. (95% CI: 9–11 p.p.)	Subgroup analysis: Among younger (aged < 45): 11 p.p. (95% CI: 9–13 p.p.) Among vaccine-ambivalent: 15 p.p. (95% CI: 14–16 p.p.) Among vaccine-hesitant: 3 p.p. (95% CI: 2–4 p.p.)
	Dining out after 8 pm	6 p.p. (95% CI: 5–7 p.p.)	Subgroup analysis: Among younger (aged < 45): 7 p.p. (95% CI: 6–8 p.p.) Among vaccine-ambivalent: 9 p.p. (95% CI: 8–10 p.p.) Among vaccine-hesitant: 3 p.p. (95% CI: 2–4 p.p.)
	Join social gatherings/events	4 p.p. (95% CI: 3–5 p.p.)	Subgroup analysis: Among younger (aged < 45): 4 p.p. (95% CI: 3–5 p.p.) Among vaccine-ambivalent: 6 p.p. (5–7 p.p.) Among vaccine-hesitant: 1 p.p. (0–2 p.p.)
	Going out without masks	7 p.p. (95% CI: 6–8 p.p.)	Subgroup analysis: Among younger (aged < 45): 7 p.p. (95% CI: 6–8 p.p.) Among vaccine-ambivalent: 10 p.p. (95% CI: 9–11 p.p.) Among vaccine-hesitant: 4 p.p. (95% CI: 3–5 p.p.)
[30]	Absolute increase in COVID-19 vaccine doses 20 days prior to the introduction of vaccine certificates (in anticipation):		Increases in vaccine doses are comparing “actual” vs. “counterfactual” estimates using 19 control countries. Denmark and Germany were analyzed, but changes were non-significant
	Israel	-31,485 (95% CI: -489,267; -31,485)	This is equivalent to -3,582 (95% CI: -55,663; -3,582) doses per million population
	Switzerland	153,152 (95% CI: 31,851–221,482)	This is equivalent to 17,572 (95% CI: 3,655–25,412) doses per million population
	Italy	2,513,065 (95% CI: 1,505,684–3,207,418)	This is equivalent to 41,629 (95% CI: 24,942–53,132) doses per million population
	France	3,761,440 (95% CI: 3,355,761–4,979,952)	This is equivalent to 55,672 (95% CI: 49,668–73,707) doses per million population
	Absolute increase in COVID-19 vaccine doses within 40 days post-introduction of vaccine certificates:		Increases in vaccine doses are comparing “actual” vs. “counterfactual” estimates using 19 control countries. Denmark and Germany were analyzed, but changes were non-significant
	Israel	2,168,728 (95% CI: 1,925,688- 2,364,362)	This is equivalent to 246,733 (95% CI: 219,083–268,990) doses per million population
	Switzerland	412,940 (95% CI: 86,021–685,270)	This is equivalent to 47,380 (95% CI: 9,870–78,627) doses per million population
	Italy	1,494,270 (95% CI: 72,366–4,475,654)	This is equivalent to 24,753 (95% CI: 1,199–74,140) doses per million population
	France	4,874,857 (95% CI: 2,563,396–7,711,769)	This is equivalent to 72,151 (95% CI: 37,940–114,140) doses per million population
[24]	Prevalence of survey respondents that cited the EU COVID certificate as the reason to receive COVID-19 vaccination (in France)	36.2% (*n* = 72/199)	In contrast, 22.6% (*n* = 45/199) of respondents cited that the EU COVID certificate did not influence willingness to vaccinate
[26]	Perceived influence of vaccine certificates for overseas travel on a sliding scale from 0 (not impactful) to 10 (most impactful) (in China)	4.44 out of 10 (95% CI: 4.18–4.71)	Vaccine certificates for overseas travel was the highest-rated facilitator to COVID-19 vaccination
[33]	Participants that cited COVID-19 vaccine certificates as a reason for receiving vaccines (in the United Kingdom)	N/A (Qualitative study)	Vaccine certificates could ‘nudge’ vaccine delayers to vaccinate for travel, work, and social purposes
[34]	Unvaccinated participants cited that the use of COVID-19 vaccine certificates resulted in a positive influence for vaccination	39%	76% of the population measured strongly supported the implementation of a vaccine passport
[35]	Increase in COVID-19 vaccine uptake with the introduction of a vaccine passport	13.98% in Poland, and 19.75% in Lithuania	3.88% of those 80 year old (vaccinated individuals) or older were convinced In unvaccinated: 26.10% of those aged 18–24, 26.89%in the 25–49 age group, 27.47% of those aged 50–59, 22.98% in the 60–69 group, and 16.89% in the 70–79 group, but only 8.14% of those aged 80 and more
[36]	Increase in COVID-19 vaccine uptake with the introduction of a vaccine passport		
	British Columbia	4.4p.p. (95% CI 2.1–6.6)	This is equivalent to to 203,300 (98,253–308,346) more people being vaccinated
	Alberta	8.2p.p. (95% CI 7.0–9.4)	This is equivalent to to 310,890 (267,169–354,611) more people being vaccinated
	Saskatchewan	7.2p.p., 95% CI 5.3–9.1)	This is equivalent to 71,711 (52,337–91,084) more people being vaccinated
	Manitoba	5.4p.p. (95% CI 4.0–6.9)	This is equivalent to 63,936 (46,841–81,030) more people being vaccinated
	Nova Scotia	5.2p.p. (95% CI 1.6–8.8)	This is equivalent to 44,054 (14,052–78,056) more people being vaccinated
	Newfoundland and Labrador	6.4p.p. (95% CI 3.8–9.0)	This is equivalent to 29,814 (17,542–42,086) more people being vaccinated
	Ontario	No significant change	
	Quebec	No significant change	
	New Brunswick	No significant change	
	Prince Edward Island	Decreased vaccine uptake^**^	
*Decreased willingness to vaccinate (n* = *1)*			
[23]	Prevalence of survey respondents who declared they may reconsider their wish to be vaccinated if national QR code-based vaccine certificates are implemented (in Russia)	26.59%	Vaccine uptake in Russia followed an exponential increase except for two major periods of slowdown (August 23–October 20, 2021; and November 25–January 15, 2022), which correspond to time periods when Russian QR code-based vaccine certificates were introduced
*Mixed findings or no effect (n* = *3)*			
[25]	Predicted impact of COVID-19 vaccine certificates on vaccine uptake (based on logistic regression analysis) (in Israel)	OR: 1.12 (95% CI: 0.82–1.55)	Vaccine certificates had a positive impact on vaccine uptake, but non-significant
[39]	Impact of public messaging about the benefits of owning COVID-19 vaccine certificates on willingness to vaccinate		
	France	OR: 0.87 (95% CI: 0.46–1.63)	Messaging had a negative impact on vaccine uptake, but non-significant
	Sweden	OR: 0.92 (95% CI: 0.66- 1.27)	Messaging had a negative impact on vaccine uptake, but non-significant
	Poland	OR: 0.97 (95% CI: 0.61–1.54)	Messaging had a negative impact on vaccine uptake, but non-significant
	Italy	OR: 0.97 (95% CI: 0.71–1.34)	Messaging had a negative impact on vaccine uptake, but non-significant
	Spain	OR: 0.97, (95% CI: 0.96–1.36)	Messaging had a negative impact on vaccine uptake, but non-significant
	Germany	OR: 1.44 (95% CI: 1.09–1.91)	Messaging had a positive impact on vaccine uptake and was significant
	United Kingdom	OR: 1.51 (95% CI: 1.11–2.05)	Messaging had a positive impact on vaccine uptake and was significant
	Bulgaria	OR: 1.58 (95% CI: 0.67- 3.73)	Messaging had a positive impact on vaccine uptake, but non-significant
[27]	Prevalence difference of survey respondents that were “inclined to accept COVID-19 vaccines” if vaccine certificates are introduced for domestic use (in the U.K.)		Responses were given on a 5-point ordinal scale: *much less inclined, somewhat less inclined, no more or less inclined, somewhat more inclined, and much more inclined*
	Much less inclined	6.41% (95% CI: 5.61–7.38%)	
	Somewhat less inclined	4.08% (95% CI: 3.66–4.54%	
	No more or less inclined	46.5% (95% CI: 44.4–48.7%)	
	Somewhat more inclined	14.9% (95% CI: 14.4–15.5%)	
	Much more inclined	28.1% (95% CI: 25.3–31.1%)	
	Prevalence difference of survey respondents that were “inclined to accept COVID-19 vaccines” if vaccine certificates are introduced for international use (in the U.K.)		Responses were given on a 5-point ordinal scale: *much less inclined, somewhat less inclined, no more or less inclined, somewhat more inclined, and much more inclined*
	Much less inclined	5.61% (95% CI: 4.86–6.34%)	
	Somewhat less inclined	3.95% (95% CI: 3.52–4.35%)	
	No more or less inclined	42.0% (95% CI: 39.8–43.9%)	
	Somewhat more inclined	14.9% (95% CI: 14.6–15.2%)	
	Much more inclined	33.6% (95% CI: 30.8–36.6%)	

^*^p.p denotes percentage-points

There three most frequently referenced countries were: France (vaccine uptake increased by 8–13 percentage-points (p.p.) associated with implementation of vaccine certificates), Germany (vaccine uptake increased by 4.7–6.2 percentage-points) associated with implementation of vaccine certificates), and Italy (vaccine uptake increased by 9.7–12 percentage-points associated with the implementation of vaccine certificates) [28–30]. Mills et al. [30] corroborate these findings for France and Italy, consistently demonstrating a statistically significant increase in COVID-19 vaccine doses at two timepoints: 20 days prior to the implementation of vaccine certificates in those countries (in anticipation of their implementation), with effects lasting up to 40 days post-implementation. In Canada [34], reported that the implementation a vaccine passport resulted in a 39% increase in vaccine uptake [34]. Maquiling et al. [36] also reported that in Canada six out of ten provinces saw a statistically significant increase in vaccination following the implementation of vaccine passports [36]. The average increase within these six provinces was found to be 6.13 p.p [36]. It was also found that the implementation of a vaccine passport resulted in increasing the vaccination rate by 13.98% in Poland, and 19.75% in Lithuania [35]. These increases were seen in the youngest age group (18–24 years of age) [35]. More detail about each age group is found in Table 5. Interestingly, this trend was inconsistent for Israel: Mills et al. [30] found a small but statistically significant decrease in vaccine uptake at the 20 days pre-implementation period, followed by a large statistically significant increase in uptake at the 40 days post-implementation period. These trends may be moderated by the population characteristics and implementation strategy of the vaccine certificates: Okamoto et al. [32]documented that vaccine certificates implemented for facilitating “travel across prefectures” was associated with the greatest increase in vaccine uptake (10 p.p. (percentage points)), followed by vaccine certificates implemented for “going out without masks” (7 p.p.), “dining out after 8 pm” (6 p.p.), and “joining social gatherings or events” (4 p.p.). In their subgroup analysis, these effects tended to be amplified among vaccine-ambivalent survey respondents (15 p.p., 10 p.p., 9 p.p., and 6 p.p., for vaccine certificate-mediated travel, going out without masks, dining, and social events, respectively), although they tended to be diminished among vaccine-hesitant survey respondents (3 p.p., 4 p.p., 3 p.p., and 1 p.p., for vaccine certificate-mediated travel, going out without masks, dining, and social event privileges, respectively).

Only one study (6.3%; *n* = 1/16) found that COVID-19 vaccine certificates significantly associated with a decrease in vaccination [23], and three studies (18.8%; *n* = 3/16) reported mixed or non-significant findings (Table 5) [25, 27, 39]. Notably, Boguslavsky et al. [23]documented that, among Russian survey respondents, 26.59% may avoid COVID-19 vaccines if QR code-based vaccine certificates were to be introduced. The primary concern was not the idea of vaccine certificates itself, but rather, the low receptivity of the Russian population to the proposed digital QR code-based system of public health surveillance: Boguslavsky et al. [23] found that approximately 94% of individuals who refused to be vaccinated and approximately 87% of their whole sample was opposed to the introduction of a QR code-based approach to digital vaccine certificates in Russia. Boguslavsky et al. [23] proposes two main reasons for this. First, there are prevalent concerns among the Russian public that QR code-based vaccine certificates will lead to potential segregation of the Russian public (those that do not have them will be “castaways” in society); denied access to shops, markets, work, various social venues, transportation, and other public and private sectors; perceived endangerment of digital privacy, lack of financial protection (especially with respect to online banking systems), and potential fraud related to QR codes; etc [23]. Second, the Russian government and media may have also fostered anti-vaccination sentiments and creating negative views of COVID-19 vaccines and vaccine certificates over two critical periods (August 23–October 20, 2021; and November 25–January 15, 2022) during which the government strongly pushed for the introduction of a QR code-based system of digital vaccine certificates and widely promoted this across Russian news media platforms [23]. However, it is important to note that the general Russian population has very low trust in their government and in turn, the high number of individuals who refused to be vaccinated point to the importance of trust and social capital as facilitators in the implementation of vaccine certificates.

## Discussion

To the best of our knowledge, this is the first scoping review that overviews the association between vaccine certificate implementation and willingness to vaccinate on a global scale and barriers and facilitators to their use. Multiple novel interventions were implemented during the pandemic and researchers have attempted to study their impact. There has been substantial variability in the quality of this research and the subsequent evidence produced [21, 41].

Given the impact of vaccine certificates and their potential for future use a scoping review provides a broad overview of the emerging literature on this topic.

In our scoping review, the majority of studies (75%; *n* = 12/16) found that vaccine certificates had a positive association on the rate of vaccine uptake across multiple countries (Table 5). This positive relationship was most commonly observed for three European countries: France, Germany, and Italy (Table 3) [24, 28–30]. Interestingly, only one study [23] in this review linked the implementation of vaccine certificates to a reduced COVID-19 vaccine uptake (Tables 3 and 5). Boguslavsky et al. [23] propose that this was primarily due to the QR code-based platform that the Russian government was planning to use for their digital COVID-19 vaccine certificates.

Our findings need to be taken into context given the heterogeneity of settings and implementation strategies for vaccine certificates. We attempted to characterize some of this heterogeneity by describing internal and external barriers and facilitators to the impact of vaccine certificates.

The intrinsic facilitators that we identified in (Table 4) reflect “carrot”-type strategies to promote vaccine uptake by disseminating information about the safety and efficacy of COVID-19 vaccines, as well as appealing to the public’s social responsibility to protect themselves, their family and friends, and society at-large. These intrinsic facilitators to vaccination were cited less frequently in our review compared to the extrinsic facilitators, although the literature emphasizes their important role in the implementation of vaccine certificates and promoting vaccine uptake. Notably, in our review, a study [39] documented that effective messaging about the safety, efficacy, and medical or hedonistic benefits of COVID-19 vaccines has the potential to mitigate vaccine-hesitant attitudes and promote vaccine uptake; although, these findings were non-significant. Steinert et al. [39] suggest that widespread conspiracy beliefs and low health literacy undermines and reduces the effect of this messaging, which could serve as future targets for public health interventions and should be considered when implementing vaccine certificate and vaccine campaigns. These findings were corroborated by other studies, which suggested that messaging and framing designed to garner increased trust in the safety/efficacy of the COVID-19 vaccines and better understanding of the potential benefits of vaccines for population health and the economy/society at-large — in other words, framing COVID-19 messaging to better appeal to the intrinsic facilitators that we identified in (Table 4)— appeared to be instrumental for the effective implementation of vaccines and vaccine certificates [42–45]. Ultimately, our findings support the existing literature about the importance of incorporating framing and messaging about these intrinsic facilitators during COVID-19 vaccine and vaccine certificate campaigns.

Second, the external facilitators that we identified in (Table 4) reflect “stick”-type strategies to promote vaccine uptake by leveraging vaccine certificates as a “gatekeeping” system to restrict access to various social, work, and travel privileges for individuals lacking proof of vaccination. Our review found that travel (both domestically and globally) and work privileges contingent upon having vaccine certificates were among the most frequently cited facilitators to COVID-19 vaccination, which is consistent with the surrounding literature [20, 46, 47]. A global survey of 23 countries reported in July 2021 that there was generally strong support for travel and work mandates contingent on proof of vaccination, with an average of 74.4% and 62.3% of respondents agreeing with requiring vaccine certificates for international travel and employment, respectively [48]. Support for these mandates was lowest in Russia (52.5% and 30.9% of Russian respondents supported travel and work mandates, respectively) [48] which is consistent with the findings in our review. Interestingly, this survey found that the three European countries for which we observed a strong positive impact of vaccine certificates on vaccine uptake — France, Germany, and Italy — had below-average support for travel and work mandates contingent on proof of vaccination: only 66.6%, 66.3%, and 73.0% of French, Germany, and Italian respondents supported travel mandates contingent upon proof of vaccination, respectively, and only 49.3%, 40.3%, and 57.6% of French, Germany, and Italian respondents supported work mandates contingent upon proof of vaccination, respectively [48].

We did not systematically examine the impact of vaccine certificate introduction on other end points. However, several of our included studies did examine the positive impact of vaccine certificate introduction on the economy and on reducing health care burden. Future studies should systematically examine the potential association of vaccine certificates on health and economic outcomes. Future studies should also explore how mechanisms of implementation affected the impact of vaccine certificates.

### Strengths and limitations

This study has numerous strengths. First, our evidence base included a wide-ranging set of study designs (observational, modeling, experimental, and qualitative studies) and was not limited to only peer-reviewed articles (our search strategy included preprint servers such as Medrxiv and Biorxiv). Second, our search strategy was not constrained to specific settings or populations, therefore, enabling us to evaluate vaccine certificate and vaccination campaigns across multiple countries at a global scale and make cross-national comparisons. Third, we evaluated the quality of the studies that employed a survey using a standard instrument.

There were also several limitations in this study. First, the pandemic created multiple natural experiments that provided an opportunity for evaluation. The variability in the quality of these evaluations limits their potential generalizability of their findings. We found, in our analysis, that the quality of the studies was generally good. However, there is substantial variability to how vaccine certificates were implemented and the local culture that contributes to their impact that limits the generalizability of our findings. We attempted to capture some aspects of this through our analysis of barriers and facilitators but there are many other confounding variables that impact the relationship between vaccine certificate implementation and vaccination rates. A general consistency of effect across multiple jurisdictions does suggest potential for improvement in vaccination rates. However, substantial heterogeneity and potential for co-interventions limits the ability to make any causal assessments. As such, the results of this review should be viewed as exploratory and hypothesis generating. Second, this review was limited to articles published in the English language. Future studies should aim to include articles published in other languages, to ensure a comprehensive evaluation of the impact of vaccine certificates on willingness to vaccinate, which is especially important given the global scale of this issue. Future studies should also systematically examine the impact of these interventions on mortality and the economy. Third, there is no standard tool for reporting on the survey studies included in our analysis [38]. Fourth, there is a potential for publication bias, where studies that showed no effect of vaccine certificates were not submitted for publication.

## Conclusion

Achieving high vaccine coverage during the COVID-19 pandemic was crucial to reducing the transmission of SARS-CoV-2 and mitigating the impact of the pandemic on the healthcare system and society at-large. Within this rapidly evolving and transitional period, the ability to track (e.g., using vaccine certificates) those who have been vaccinated, versus those who refused or delayed vaccination, was potentially valuable for governments and public health officials to make evidence-based policy decisions about how to safely return society to normalcy. However, this approach has not been without controversy and had potential negative effects. Our scoping review provides insights about the various facilitators and barriers to COVID-19 vaccination related to vaccine certificates, as well as an overview of the observed impacts of vaccine certificates on COVID-19 vaccine uptake across multiple countries. These findings reflect important considerations for future implementation of vaccine certificates for later stages of the current pandemic as well as other emergent public and global health threats.

### Supplementary Information

**Additional file 1.** 

**Additional file 2.**

## Data Availability

Not applicable.

## Appendix 1

Search Terms Used

Embase Classic + Embase < 1947 to 2023 July 07 > 

Ovid MEDLINE(R) ALL < 1946 to July 06, 2023 > 

APA PsycInfo < 1806 to July Week 1 2023 > 

Medline.

1 (exp coronavirus/ or coronavirus*.mp. or corona virus*.mp.) and (wuhan or beijing or shanghai).mp. 20,182.

2 ((coronavirus or corona virus) adj3 "2019").tw. 139,185.

3 (covid or covid2019).tw,kf. 766,524.

4 covid19.tw,kw. or covid 19.kf. 351,973.

5 sars cov 2.tw,kw. 270,410.

6 (ncov or n cov).tw,kw. 7183.

7 novel coronavirus.tw,kw. 28,681.

8 sars cov2.tw,kw. 12,300.

9 Coronavirus Infections/ and Pandemics/ 46,292.

10 COVID-19 Vaccines/ 47,031.

11 (ncov19 or ncov-19 or 2019-novel CoV).tw,kf. 2770.

12 or/1–11 841,947.

13 (exp Vaccination/ or exp Immunization/ or exp Immunization Programs/) and documentation/ 1046.

14 passport*.tw,kf. 3811.

15 ((immunity or immune or immuni?ation or vaccin*) adj5 (certificat* or document* or proof)).tw,kf. 9808.

16 ((immunity or immune or immuni?ation or vaccin*) adj2 (mandate* or require*)).tw,kf. 15,401.

17 or/13–16 29,239.

18 12 and 17 3343.

19 "patient acceptance of health care"/ or patient compliance/ 326,844.

20 Vaccination Refusal/ 2117.

21 (uptake or hesitan* or complian* or accept* or attitude*).tw,kf. 3,541,681.

22 (vaccin* adj2 refus*).tw,kf. 3737.

23 vaccin* confidence.tw,kf. 1502.

24 or/19–23 3,761,891.

25 18 and 24 959.

26 25 use medall 422.

27 limit 26 to dt = 20,220,513–20230710 237.

Embase.

28 coronavirus disease 2019/ 599,261.

29 (Coronavirinae/ or coronavirus*.mp. or corona virus*.mp.) and (wuhan or beijing or shanghai or hubei).mp. 20,232.

30 ((coronavirus* or corona virus* or coronavirus* or coronaviridae or coronaviridae or betacoronavirus*) adj3 ("19" or "2019")).tw. 171,401.

31 (covid or covid19 or covid2019).tw. 741,571.

32 sars cov 2.tw. 240,295.

33 (ncov or n cov).tw. 7154.

34 (novel coronavirus* or novel corona virus*).tw. 28,800.

35 (CoV 2 or CoV2 or sarscov2 or 2019nCoV or novel CoV or wuhan virus).tw. 247,843.

36 exp SARS-CoV-2 vaccine/ 60,849.

37 or/28–36 867,789.

38 ((immunity or immune or immuni?ation or vaccin*) adj5 (certificat* or document*)).tw. 8356.

39 passport*.mp. 3912.

40 "immunity passport"/ 6.

41 ((immunity or immune or immuni?ation or vaccin*) adj5 (certificat* or document* or proof)).tw. 9787.

42 ((immunity or immune or immuni?ation or vaccin*) adj2 (mandate* or require*)).tw. 15,367.

43 or/38–42 28,638.

44 37 and 43 3263.

45 vaccine hesitancy/ 7392.

46 patient attitude/ or patient compliance/ 301,678.

47 (vaccin* adj2 refusal).tw. 1838.

48 (uptake or hesitan* or complian* or accept* or attitude*).tw. 3,512,166.

49 vaccine confidence.tw. 1175.

50 or/45–49 3,717,501.

51 44 and 50 930.

52 51 use emczd 495.

53 limit 52 to dc = 20,220,516–20230710 311.

PsycInfo.

54 covid-19/ 437,209.

55 (covid or covid19 or covid2019 or sars cov 2).tw.809142.

56 ((coronavirus or corona virus) adj3 "2019").tw. 139,185.

57 (ncov or n cov).tw. 7154.

58 novel coronavirus.tw. 27,673.

59 or/54–58 846,511.

60 immunization/ and (certificat* or document* or proof).tw. 4200.

61 passport*.tw. 3689.

62 ((immunity or immune or immuni?ation or vaccin*) adj5 (certificat* or document* or proof)).tw. 9787.

63 ((immunity or immune or immuni?ation or vaccin*) adj2 (mandate* or require*)).tw. 15,367.

64 or/60–63 31,392.

65 59 and 64 3369.

66 treatment compliance/ or compliance/ 248,505.

67 (vaccin* adj2 refusal).tw. 1838.

68 (uptake or hesitan* or complian* or accept* or attitude*).mp. 4,780,667.

69 vaccine confidence.tw. 1175.

70 or/66–69 4,781,242.

71 65 and 70 987.

72 71 use psyh 45.

73 limit 72 to up = 20,220,507–20230710 27.

74 27 or 53 or 73 575.

75 remove duplicates from 74 367.

Medrxiv (via Google Scholar).

2022—2023 -21 References.

(source:medrxiv) AND (COVID OR COVID19 OR COVID2019 OR Sars Cov 2 OR Novel Coronavirus) AND (passport* OR document* OR mandate*) AND (uptake OR hesitancy OR compliance OR accept OR acceptance OR attitude).

https://scholar.google.com/scholar?q=%28source%3Amedrxiv%29+AND+%28COVID+OR+COVID+19+OR+COVID+2019+OR+Sars+Cov+2+OR+Novel+Coronavirus%29+AND+%28passport*+OR+document*+OR+mandate*%29+AND+%28uptake+OR+hesitancy+OR+compliance+OR+accept+OR+acceptance+OR+attitude%29&hl=en&as_sdt=0%2C5&as_ylo=2022&as_yhi=2023

Biorxiv (via Google Scholar).

2022 -2023 – 2 References.

(source:bioRxiv) AND (COVID OR COVID19 OR COVID2019 OR Sars Cov 2 OR Novel Coronavirus) AND (passport* OR document* OR mandate*) AND (uptake OR hesitancy OR compliance OR accept OR acceptance OR attitude) https://scholar.google.com/scholar?q=%28source%3AbioRxiv%29+AND+%28COVID+OR+COVID+19+OR+COVID+2019+OR+Sars+Cov+2+OR+Novel+Coronavirus%29+AND+%28passport*+OR+document*+OR+mandate*%29+AND+%28uptake+OR+hesitancy+OR+compliance+OR+accept+OR+acceptance+OR+attitude%29&hl=en&as_sdt=0%2C5&as_ylo=2022&as_yhi=2023

LOVE Platform – July 10, 2023 https://app.iloveevidence.com/loves/5e6fdb9669c00e4ac072701d?population=5e7fce7e3d05156b5f5e032a&intervention_variable=603b9fe03d05151f35cf13dc&classification=all

((vaccin* OR vaccination* OR immunisation* OR immunization*)) AND ((passport* OR document* OR mandate*)) AND ((uptake or hesitanc* or compliance* or accept OR acceptance or attitude*)).

Limited to preprints: 32 References.

## Appendix 2

PRISMA-ScR Checklist

## Abbreviation

COVID-19: Coronavirus Disease 2019
