# Supplementary material for: The barriers, facilitators and association of vaccine certificates on COVID-19 vaccine uptake: a scoping review
Source: Global Health. 2023 Sep 27;19:73. doi: 10.1186/s12992-023-00969-y (PMC10537206; doi:10.1186/s12992-023-00969-y)
Supplement: Supplementary file 1 — Additional file 1. [file 12992_2023_969_MOESM1_ESM.docx]

Embase Classic+Embase <1947 to 2022 May 16>

Ovid MEDLINE(R) ALL <1946 to May 13, 2022>

APA PsycInfo <1806 to May Week 2 2022>

1 (exp coronavirus/ or coronavirus*.mp. or corona virus*.mp.) and (wuhan or beijing or shanghai).mp. 14896

2 ((coronavirus or corona virus) adj3 "2019").tw. 95852

3 (covid or covid2019).tw,kf. 483288

4 covid19.tw,kw. or covid 19.kf. 222413

5 sars cov 2.tw,kw. 172013

6 (ncov or n cov).tw,kw. 4985

7 novel coronavirus.tw,kw. 22805

8 sars cov2.tw,kw. 8035

9 Coronavirus Infections/ and Pandemics/ 48252

10 COVID-19 Vaccines/ 23713

11 (ncov19 or ncov-19 or 2019-novel CoV).tw,kf. 1507

12 or/1-11 531594

13 (exp Vaccination/ or exp Immunization/ or exp Immunization Programs/) and documentation/ 913

14 passport*.tw,kf. 3336

15 ((immunity or immune or immuni?ation or vaccin*) adj5 (certificat* or document* or proof)).tw,kf. 8714

16 ((immunity or immune or immuni?ation or vaccin*) adj2 (mandate* or require*)).tw,kf. 13471

17 or/13-16 25748

18 12 and 17 1672

19 "patient acceptance of health care"/ or patient compliance/ 314657

20 Vaccination Refusal/ 1672

21 (uptake or hesitan* or complian* or accept* or attitude*).tw,kf. 3283916

22 (vaccin* adj2 refus*).tw,kf. 2899

23 vaccin* confidence.tw,kf. 903

24 or/19-23 3495922

25 18 and 24 419

**26** **25 use medall** **191**

**27** **limit 26 to dt=20220120-20220517** **64**

**Embase**

28 coronavirus disease 2019/ 374151

29 (Coronavirinae/ or coronavirus*.mp. or corona virus*.mp.) and (wuhan or beijing or shanghai or hubei).mp. 15193

30 ((coronavirus* or corona virus* or coronavirus* or coronaviridae or coronaviridae or betacoronavirus*) adj3 ("19" or "2019")).tw. 118744

31 (covid or covid19 or covid2019).tw. 467662

32 sars cov 2.tw. 151698

33 (ncov or n cov).tw. 4960

34 (novel coronavirus* or novel corona virus*).tw. 22908

35 (CoV 2 or CoV2 or sarscov2 or 2019nCoV or novel CoV or wuhan virus).tw. 156641

36 exp SARS-CoV-2 vaccine/ 28387

37 or/28-36 549187

38 ((immunity or immune or immuni?ation or vaccin*) adj5 (certificat* or document*)).tw. 7450

39 passport*.mp. 3433

40 "immunity passport"/ 6

41 ((immunity or immune or immuni?ation or vaccin*) adj5 (certificat* or document* or proof)).tw. 8701

42 ((immunity or immune or immuni?ation or vaccin*) adj2 (mandate* or require*)).tw. 13448

43 or/38-42 25236

44 37 and 43 1623

45 vaccine hesitancy/ 3450

46 patient attitude/ or patient compliance/ 289933

47 (vaccin* adj2 refusal).tw. 1392

48 (uptake or hesitan* or complian* or accept* or attitude*).tw. 3257653

49 vaccine confidence.tw. 676

50 or/45-49 3454433

51 44 and 50 402

**52** **51 use emczd** **209**

**53** **limit 52 to dc=20220120-20220517** **96**

**PsycInfo**

54 covid-19/ 216466

55 (covid or covid19 or covid2019 or sars cov 2).tw. 508859

56 ((coronavirus or corona virus) adj3 "2019").tw. 95852

57 (ncov or n cov).tw. 4960

58 novel coronavirus.tw. 21990

59 or/54-58 531388

60 immunization/ and (certificat* or document* or proof).tw. 3791

61 passport*.tw. 3249

62 ((immunity or immune or immuni?ation or vaccin*) adj5 (certificat* or document* or proof)).tw. 8701

63 ((immunity or immune or immuni?ation or vaccin*) adj2 (mandate* or require*)).tw. 13448

64 or/60-63 27735

65 59 and 64 1653

66 treatment compliance/ or compliance/ 238699

67 (vaccin* adj2 refusal).tw. 1392

68 (uptake or hesitan* or complian* or accept* or attitude*).mp. 4476036

69 vaccine confidence.tw. 676

70 or/66-69 4476500

71 65 and 70 421

**72** **71 use psyh** **18**

**73** **limit 72 to up=20220120-20220517** **7**

74 27 or 53 or 73 167

**Medrxiv (via Google Scholar)**

May 17, 2022 -31 References

(source:medrxiv) AND (COVID OR COVID19 OR COVID2019 OR Sars Cov 2 OR Novel Coronavirus) AND (passport* OR document* OR mandate*) AND (uptake OR hesitancy OR  compliance OR  accept OR acceptance OR attitude) 
<https://scholar.google.com/scholar?hl=en&as_sdt=2005&sciodt=0%2C5&cites=15538255288147389482&scipsc=&q=%28source%3Amedrxiv%29+AND+%28COVID+OR+COVID19+OR+COVID2019+OR+Sars+Cov+2+OR+Novel+Coronavirus%29+AND+%28passport*+OR+document*+OR+mandate*%29+AND+%28uptake+OR+hesitancy+OR++compliance+OR++accept+OR+acceptance+OR+attitude%29&btnG=>

**Biorxiv (via Google Scholar)**

May 17, 2022 – 2 References

(source:bioRxiv) AND (COVID OR COVID19 OR COVID2019 OR Sars Cov 2 OR Novel Coronavirus) AND (passport* OR document* OR mandate*) AND (uptake OR hesitancy OR  compliance OR  accept OR acceptance OR attitude)

**LOVE Platform** – May 17, 2022

<https://app.iloveevidence.com/loves/5e6fdb9669c00e4ac072701d?population=5e7fce7e3d05156b5f5e032a&intervention_variable=603b9fe03d05151f35cf13dc&classification=all>

((vaccin* OR vaccination*  OR immunisation* OR immunization*)) AND ((passport* OR document* OR mandate*)) AND ((uptake or hesitanc* or compliance* or accept OR acceptance or attitude*))

Limited to preprints: 69 References
