# Supplementary material for: The barriers, facilitators and association of vaccine certificates on COVID-19 vaccine uptake: a scoping review
Source: Global Health. 2023 Sep 27;19:73. doi: 10.1186/s12992-023-00969-y (PMC10537206; doi:10.1186/s12992-023-00969-y)
Supplement: Supplementary file 2 — Additional file 2. [file 12992_2023_969_MOESM2_ESM.docx]

# Eligibility criteria

| **The following articles were included:** |
| --- |
| Examined the general adult population rather than special/vulnerable populations  Included discussion of COVID-19 vaccine certificate characteristics (or synonyms such as immunity passports, green passes, proof of vaccination, etc.)  Included discussion of participants’ willingness to receive COVID-19 vaccines (acceptance, delay, ambivalence, hesitancy, etc.)  Evaluated the potential role of COVID-19 vaccine certificate on willingness to vaccinate  English language full text available  Primary research (e.g., observational, modeling, experimental, and qualitative studies) |
| **The following articles were excluded:** |
| No discussion of COVID-19 vaccine certificates (e.g., studies that broadly mentioned “vaccine mandates” without specifying vaccine certificates or synonyms)  No discussion of COVID-19 vaccine intention or uptake  Studies that generally described public opinions and attitudes on COVID-19 certificates and/or hesitancy but did not evaluate their interaction/association  No English language full text available  Review-type or non-empirical studies (e.g., commentaries, editorials, opinion letters, etc.) |
